# Consolidation and translation regulation

**DOI:** 10.1101/lm.026849.112

**Published:** 2012-09

**Authors:** Shunit Gal-Ben-Ari, Justin W. Kenney, Hadile Ounalla-Saad, Elham Taha, Orit David, David Levitan, Iness Gildish, Debabrata Panja, Balagopal Pai, Karin Wibrand, T. Ian Simpson, Christopher G. Proud, Clive R. Bramham, J. Douglas Armstrong, Kobi Rosenblum

**Affiliations:** 1 Sagol Department of Neurobiology, University of Haifa, Haifa 31905, Israel; 2 Center for Gene Manipulation in the Brain, University of Haifa, Haifa 31905, Israel; 3 School of Biological Sciences, University of Southampton, Southampton SO17 1BJ, United Kingdom; 4 Department of Biomedicine and KG Jebsen Centre for Research on Neuropsychiatric Disorders, University of Bergen, 5009 Bergen, Norway; 5 School of Informatics, University of Edinburgh, Edinburgh EH8 9AB, United Kingdom

## Abstract

mRNA translation, or protein synthesis, is a major component of the transformation of the genetic code into any cellular activity. This complicated, multistep process is divided into three phases: initiation, elongation, and termination. Initiation is the step at which the ribosome is recruited to the mRNA, and is regarded as the major rate-limiting step in translation, while elongation consists of the elongation of the polypeptide chain; both steps are frequent targets for regulation, which is defined as a change in the rate of translation of an mRNA per unit time. In the normal brain, control of translation is a key mechanism for regulation of memory and synaptic plasticity consolidation, i.e., the off-line processing of acquired information. These regulation processes may differ between different brain structures or neuronal populations. Moreover, dysregulation of translation leads to pathological brain function such as memory impairment. Both normal and abnormal function of the translation machinery is believed to lead to translational up-regulation or down-regulation of a subset of mRNAs. However, the identification of these newly synthesized proteins and determination of the rates of protein synthesis or degradation taking place in different neuronal types and compartments at different time points in the brain demand new proteomic methods and system biology approaches. Here, we discuss in detail the relationship between translation regulation and memory or synaptic plasticity consolidation while focusing on a model of cortical-dependent taste learning task and hippocampal-dependent plasticity. In addition, we describe a novel systems biology perspective to better describe consolidation.

## Regulation of mRNA translation

The control of mRNA translation plays a critical role in regulating protein production; indeed, its contribution is greater than those of the modulation of mRNA synthesis, degradation, or protein turnover (Schwanhausser et al. 2011). In particular, controlling mRNA translation, rather than mRNA levels, allows protein synthesis to be regulated spatially at defined locations within the cell, and temporally, in response to particular stimuli or conditions. This article will focus principally on mechanisms that are implicated in controlling protein synthesis in neurons, especially postsynaptically.

### Regulation of translation initiation

**Figure 1.**
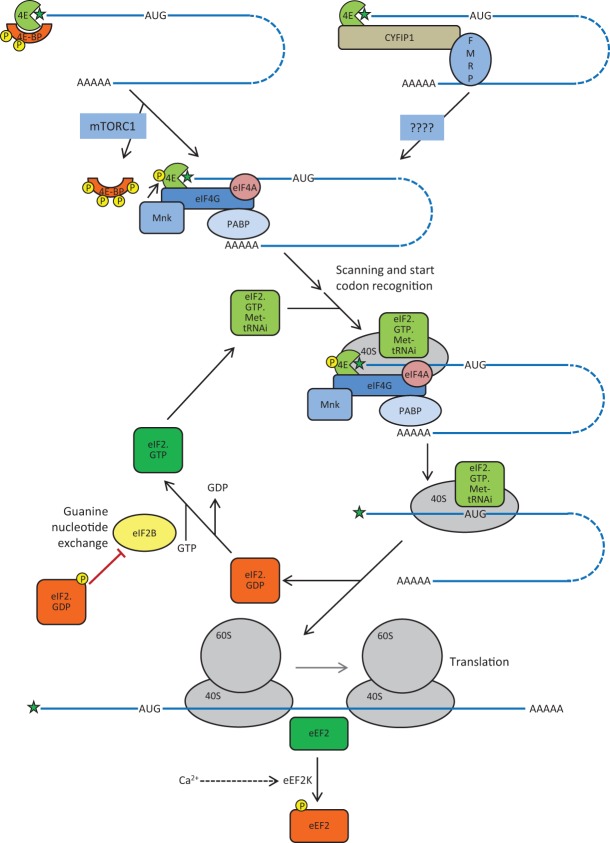
Schematic depiction of the steps in mRNA translation that are discussed in the text, including the recruitment of eIF4E to the 5′-cap (star) of the mRNA (blue line). Phosphoryation events are indicated by “P” in a yellow circle. Following phosphorylation of 4E-BP1 by mTORC1, eIF4E is able to bind eIF4G and associated factors, leading to recruitment of the 40S ribosomal subunit and associated eIF2·GTP·Met-tRNA_i_ to the mRNA. After scanning and location of the start codon, eIF2-bound GTP is hydrolyzed to GDP, the 60S subunit joins and elongation can commence. eIF2·GDP is recycled to eIF2·GTP by eIF2B. During elongation, eEF2 mediates movement (“translocation”) of the ribosome along the mRNA; phosphorylation of eEF2, catalyzed by the Ca^2+^-activated eEF2 kinase (eEF2K), inactivates it, slowing elongation.

Eukaryotic mRNAs contain a so-called cap structure at their 5′ end that includes a 7-methylguanosine moiety linked by a 5′–5′ bond to the first nucleotide of the mRNA proper. This feature binds to eukaryotic initiation factor eIF4E and provides, in this sense, the first contact between the mRNA and components of the translational machinery (Gingras et al. 1999). eIF4E in turn binds eIF4G, a scaffold protein, which binds to the poly(A)-binding protein, PABP, and eIF4A, an RNA helicase. The association of eIF4G with proteins that bind the 5′ and 3′ ends of the mRNA (eIF4E and PABP, respectively) circularizes the mRNA, enhancing its translation. eIF4A can unwind secondary structures in the 5′ untranslated region of the mRNA (i.e., between the cap and the start codon) which otherwise impede translation. The complex containing eIF4E, eIF4A, and eIF4G is often referred to as eIF4F. eIF4G also binds the multimeric factor eIF3 which, in turn, recruits the 40S ribosomal subunit to the mRNA (Fig. 1).

eIF4E also binds other partner proteins which, since they interact with eIF4E through a site that overlaps its binding site for eIF4G, prevent eIF4E/eIF4G binding. These include the small phosphoproteins termed eIF4E-binding proteins (4E-BPs), of which 4E-BP2 is the main isoform in the brain (Bidinosti et al. 2010). 4E-BPs can be regulated by phosphorylation catalyzed by mammalian target of rapamycin complex 1, mTORC1, which results in decreased affinity of 4E-BPs for eIF4E and their release, freeing eIF4E to bind to eIF4G. Binding of 4E-BP2 can also be modulated by deamidation (Bidinosti et al. 2010). eIF4E can bind other proteins including CYFIP1, a partner for the fragile X mental retardation protein, FMRP (Napoli et al. 2008). FMRP is an RNA-binding protein that can modulate the localization, translation, and/or stability of certain mRNAs. Absence of FMRP leads to fragile X syndrome, which is associated with defective synapse maturation (De Rubeis et al. 2012).

By binding to eIF4E, CYFIP1 likely represses the translation of mRNAs that interact with FMRP; consistent with this idea, depletion of CYFIP1 leads to increased levels of proteins that are encoded by mRNAs that interact with FMRP (Napoli et al. 2008). The eIF4E/CYFIP1 interaction is disrupted following synaptic stimulation, providing a potential mechanism for controlling the synthesis of specific proteins.

eIF4E is itself subject to phosphorylation at a single site (Ser209), catalyzed by the MNKs, a small family of protein kinases, some of which are regulated by MAP kinase signaling (Buxade et al. 2008). MNK1 recognizes eIF4E through binding eIF4G. The significance of phosphorylation of eIF4E for its role in mRNA translation remains unclear; it appears to decrease the affinity of eIF4E for capped mRNA (Buxade et al. 2008).

The start codon in the mRNA is recognized by the anticodon of the specialized initiator methionyl-tRNA (Met-tRNA^Met^_i_). This tRNA is brought to the small ribosomal subunit by eIF2, which can only bind Met-tRNA^Met^_i_ when in its GTP-bound state; since eIF2-bound GTP is hydrolyzed during initiation, and eIF2 releases bound GDP only very slowly, a guanine nucleotide-exchange factor (GEF), eIF2B, is required to regenerate active eIF2·GTP.

eIF2 undergoes regulated phosphorylation at Ser51 in its α-subunit; this is important in the control of protein synthesis, since eIF2 phosphorylated at this site acts as a potent competitive inhibitor of eIF2B, blocking the regeneration of active eIF2·GTP. Mammalian cells possess four distinct eIF2 kinases, PKR, PERK, GCN2, and HRI, which are activated under different stress conditions. PERK, for example, is activated during endoplasmic reticulum stress, while GCN2 is activated by “uncharged” tRNA, which may accumulate during amino acid shortage (Wek et al. 2006). Interestingly, GCN2 helps regulate hippocampal synaptic plasticity (Costa-Mattioli et al. 2005) and also plays a role in food choice, e.g., with respect to the intake of essential amino acids (Dever and Hinnebusch 2005; Hao et al. 2005). The decreased availability of active eIF2 impairs overall protein synthesis but can actually promote the translation of some mRNAs by virtue of upstream open reading frames (uORFS) in their 5′ UTRs (Wek et al. 2006).

In the case of such mRNAs, the ribosome translates the first, most 5′, uORF, but after reaching its stop codon appears to remain on the mRNA instead of dissociating from it. It then seeks further, more 3′, start codons, for which it needs to reacquire eIF2·GTP·Met-tRNA^Met^_i_. Under normal levels of this complex, this happens rather quickly and the ribosome translates one of the next uORFs, but then quits, so it does not reach the start of the main open reading frame of the mRNA and this protein is not made. However, when eIF2 is phosphorylated and levels of eIF2·GTP·Met-tRNA^Met^_i_ are low, ribosomes will sometimes pass over the remaining uORF before acquiring the initiator tRNA. If they do so before reaching the start of the main ORF, this will now be translated, and synthesis of this protein will increase. This mechanism applies, for example, to the mRNA encoding ATF4, a transcriptional regulator that promotes transcription of genes for proteins, which help cells to deal, for example, with unfolded proteins (Palam et al. 2011).

### Regulation of translation elongation

During the elongation stage of translation the new polypeptide is assembled. This process can be regulated through the phosphorylation of elongation factors, including eukaryotic elongation factor eEF2, the protein that facilitates the movement of the ribosome along the mRNA. eEF2 is phosphorylated and inactivated by a specific protein kinase, eEF2 kinase (eEF2K), which belongs to the small family of atypical protein kinases known as α-kinases (Proud 2007). Its activity is normally strictly dependent upon Ca^2+^ ions and calmodulin (CaM). The interplay between eEF2, neurotransmitters, and ribosomal proteins is discussed in detail below.

### Regulation of translation by miRNA

In recent years microRNAs (miRNA) have been recognized as major regulators of specific protein synthesis in neurons. miRNAs are short noncoding RNAs (∼22 nt) that bind to complementary sites on the 3′ UTR of target mRNAs, where they act to inhibit protein synthesis (Filipowicz et al. 2008; Djuranovic et al. 2011; Huntzinger and Izaurralde 2011). In the canonical biogenesis pathway, miRNAs are transcribed as long primary transcripts and processed in the nucleus by the RNase III enzyme Drosha to generate a stem-loop structured precursor. The pre-miRNA is then exported to the cytoplasm where a second RNase III enzyme, Dicer, generates a mature double-stranded miRNA intermediate. One of these strands, the guide strand, is incorporated into an Argonaute protein-containing complex known as the miRNA-induced silencing complex (miRISC) (Bartel 2004). Once assembled on target bound miRNA, the RISC inhibits protein synthesis by repressing translation or promoting mRNA decay (Filipowicz et al. 2008; Huntzinger and Izaurralde 2011).

The mechanism of the miRNA-induced silencing is a subject of intensive debate (Chekulaeva et al. 2011; Fabian et al. 2011; Hafner et al. 2011). A core RISC protein for translation repression and decay is GW182 (aka TNRC6), which directly binds Ago. Evidence suggests that GW182 competes with PABP for binding to eIF4G, thereby impeding mRNA circularization and reducing translational efficiency. GW182 can also recruit CNOT1, a scaffold for recruitment of the CCR4-NOT deadenylase complex. Once deadenylated, transcripts are rapidly exposed to exonucleolytic degradation. A major issue is the relative contribution of translational repression vs. mRNA decay, and how this might function in neuronal regulation and plasticity.

## Molecular memory consolidation

Memory, which is measured as a persistent alteration in behavior is subserved by physiological and molecular mechanisms thought to be evolutionary conserved across invertebrates and vertebrates. Memory and its cellular correlates (e.g., long-term potentiation [LTP] or long-term depression [LTD]) are not unitary processes, but are comprised of phases, defined both by a temporal scale and molecular characteristics. Temporally, memory can be divided into short- and long-term phases; short-term memory (STM) lasts from minutes to hours, whereas long-term memory (LTM) lasts from days to a lifetime. On the molecular level, both short-term memory and early LTP/LTD are dependent on post-translation modifications of preexisting proteins, whereas long-term memory and long-lasting LTP/LTD are dependent on RNA transcription or protein translation (Kandel 2001; Malenka and Bear 2004). The molecular mechanisms of memory can be studied in the whole animal following training with the proper behavior and it can also be studied using reduced preparations: hippocampal slice preparation for inducing LTP or LTD in vertebrates (Malenka and Bear 2004) and sensory-motor culture of *Aplysia* for inducing long-term facilitation (LTF) in invertebrates (Kandel 2001). Although reduced preparations cannot recapture the whole spectrum of changes following behavior, they do share basic principles of cellular mechanisms.

The process in which memory becomes long lasting is termed consolidation, temporally defined by decreasing vulnerability of the memory trace to various interferences, including additional learning, seizures, cooling, neuronal inactivation, and molecular perturbation (Alberini 2009). Currently, the term consolidation or off-line processing of a given memory consists of two distinct processes: aspects of molecular consolidation, completed within hours after training, and system consolidation, taking several days or more, and thought to involve reorganization of brain circuits. However, the relationship between the two types of consolidation is poorly understood (Gildish et al. 2012).

Evidence for the requirement of transcription for memory consolidation comes from many studies in both vertebrate and invertebrate systems, which found an increase in newly synthesized RNA following learning, using labeled RNA precursors and RNA synthesis inhibitors (Matthies 1989; Alberini 2009). Transcription during memory consolidation is a very dynamic and complex process composed of phases dependent upon the type of learning involved. Usually the most prominent phase takes place immediately following training, but in some instances there are waves at 3–6 and 24 h following training (Bekinschtein et al. 2010). During these phases many types of mRNAs are transcribed (Levenson et al. 2004), such as the transcription factors *C*/*EBPβ*, *c*-*Fos*, *Zif268* (Alberini 2009), and the effector genes *Arc* (Bramham et al. 2010), *BDNF*, and *Homer1a* (Miyashita et al. 2008).

As with transcription, the involvement of translation in memory consolidation was determined following many experiments in both vertebrate and invertebrate systems. The production of newly synthesized proteins was shown following training, and its necessity for memory consolidation was established by the ability of protein synthesis inhibitors to block memory formation (Davis and Squire 1984; Matthies 1989; Sutton and Schuman 2006). As with RNA synthesis, protein synthesis following training is composed of phases, beginning immediately following training and lasting a few hours (Matthies 1989; Belelovsky et al. 2009) and in some instances days (Bekinschtein et al. 2010). Many proteins synthesized following learning are products of mRNAs that are also transcribed. However, it was shown that protein synthesis during memory formation and its cellular correlates, LTP or LTD, can be independent of new RNA synthesis (Sutton and Schuman 2006; Costa-Mattioli et al. 2009). Translation regulation in neurons adds a spatial dimension to memory consolidation, as proteins may be synthesized only in a subset of synapses (Sutton and Schuman 2006).

Until recent years, memory was viewed as a static change in synaptic function that has to be consolidated only once, and once consolidated it is resistant to destabilization. Accumulated data from the last few years have changed this perception, as recent data demonstrate that memory formation is a highly dynamic process that is prone to molecular perturbation even days or weeks following acquisition (Sacktor 2011). Moreover, they suggest that memory has to undergo several rounds of consolidation to overcome innate cellular processes that push it toward a point of reset (Wang et al. 2006; Dudai 2009; Bekinschtein et al. 2010).

## Translation and implications for human neurological disorders

Understanding how protein translation is regulated during memory consolidation is not only integral to our understanding of the molecular basis of long-term memory formation, but is also increasingly recognized as a source of potential therapeutic targets in the treatment of a variety of cognitive disorders. Dysregulation of mTORC1 has been implicated in several inherited genetic disorders that result in mental retardation. For example, a decrease in mTORC1 signaling precedes behavioral deficits in a mouse model of Rett's syndrome (Ricciardi et al. 2011) and increased mTORC1 signaling is observed both in mouse models and in the brains of human patients with fragile X syndrome (Sharma et al. 2010; Hoeffer et al. 2012). Alterations in translational regulation have also been implicated in the neuropathology and treatment of neurodegenerative disorders such as Alzheimer's and Parkinson's disease. In Alzheimer's disease, mTORC1 has been implicated in the development of neurofibrillary tangles, and signaling through mTORC1 is stimulated by amyloid β plaques that are thought to be central to Alzheimer's neuropathology, whereas there are decreases in total levels of eEF2 in the brains of those afflicted with the disease (Pei and Hugon 2008). Furthermore, in an animal model of Parkinson's disease in which mice are treated with L-DOPA, currently the most effective anti-Parkinsonian medication, inhibition of mTORC1 via rapamycin prevents the undesirable side effect of dyskinesia that is typically associated with this treatment (Santini et al. 2009). Finally, mTORC1 has also been implicated in addiction, as the administration of both psychostimulants and alcohol increases mTORC1 pathway signaling in brain regions involved in addiction, and interfering with this signaling via the administration of rapamycin prevents the reinforcing effects of these drugs (Neasta et al. 2010; Dayas et al. 2012). Thus, understanding how the translational machinery alters synaptic plasticity and neuronal function will likely have broad implications in our understanding and treatment of numerous diseases that involve cognitive dysfunction.

## Translation control by neurotransmitters

In neurons, the translation machinery is controlled not only by stress or growth signals but also by neurotransmission. However, the involvement of neurotransmitters in regulation of the translation machinery is not well understood. For example, activation of the muscarinic acetylcholine receptors has been shown to increase the expression of phosphorylated ribosomal protein S6K, eIF4E, ERK, and mTOR, suggesting stimulation of protein synthesis (Deguil et al. 2008).

Little is known about 5-hydroxytryptamine (serotonin, 5-HT) and norepinephrine and their modulation of the translation machinery. In *Aplysia-*dissociated sensory neurons, application of 5-HT results in a rapid decrease in eEF2 phosphorylation (p-eEF2) in a rapamycin-sensitive manner in neurites but an increase in p-eEF2 at the soma (Carroll et al. 2004; Weatherill et al. 2011). In contrast, chronic administration of the selective serotonin reuptake inhibitor, fluoxetine, induces eEF2 and eIF4E phosphorylation in the prefrontal cortex (PFC), hippocampus, and dentate gyrus in rats (Dagestad et al. 2006). Norepinephrine was shown to modulate monocarboxylate transporter 2 (MCT2) protein expression in cultured cortical neurons via the activation of the mTOR/S6K pathway (Chenal and Pellerin 2007).

Previous reports have shown that injections of the dopamine D1 receptor agonist SKF38393 locally to the auditory cortex of gerbils after conditioning of linear frequency-modulated tones (FMs) discrimination paradigm induces memory consolidation, and this effect is sensitive to mTORC1 inhibitors (Schicknick et al. 2008). Stimulation by dopamine of primary cortical mouse neurons causes a small increase in the phosphorylation of S6K. However, stimulation of dopamine together with both glutamate and NMDA increased S6K phosphorylation more than either reagent alone (Lenz and Avruch 2005). Whether dopamine receptors can modulate other pathways of protein synthesis, such as the initiation phase of protein translation via eIF2 or elongation phase via eEF2, is unclear.

The glutamatergic NMDA receptor (NMDAR) is involved in a variety of processes in the CNS, including synaptogenesis and synaptic plasticity (Riedel et al. 2003; Collingridge et al. 2004; Hunt and Castillo 2012). Recent studies demonstrate the importance of NMDAR signaling for mediating different pathways of the translational machinery. Distinct types of glutamate receptors, such as NMDAR, AMPAR, and mGluRs are required for activation of mTOR/S6K signaling and CaMKІІα synthesis in hippocampal dendrites following LTP (Cammalleri et al. 2003; Gong et al. 2006).

Short physiological glutamatergic stimulus in primary cortical neurons induces activation of ERK and mTOR/S6K via calcium/CaM signaling controlled by voltage-dependent calcium channels and is NMDAR independent. Continuous glutamatergic stimulus triggers high-calcium influx, leading to a progressive increase in eEF2 phosphorylation and inhibition of translation as shown in cortical neurons, cultured retinal cells of chicks, and in hippocampal slices from rats (Marin et al. 1997; Scheetz et al. 2000; Belelovsky et al. 2005; Lenz and Avruch 2005; Cossenza et al. 2006; Maus et al. 2006). The regulation of eEF2 by glutamate receptors is discussed in further detail below.

Metabotropic glutamate receptors are important for LTD induction. Stimulation of the mGluR5 receptors by dihydroxyphenylglycine (DHPG) leads to LTD, which requires postsynaptic translation of preexisting dendritically localized mRNA (Huber et al. 2001). Recent reports have shown that mGluR1 leads to increased activation of the mTOR/S6K pathway via ERK in both hippocampus and striatum (Page et al. 2006). However, a recent study has shown that S6K is not required for the mGluR-LTD (Antion et al. 2008a). In addition, DHPG treatment leads to AMPAR endocytosis via dendritic microtubule-associated protein 1B (MAP1B) up-regulation, and interestingly this MAP1B up-regulation during this treatment depends on the presence of eEF2K (Davidkova and Carroll 2007).

The regulation of eEF2 phosphorylation in response to glutamate receptor agonists and synaptic activity has been examined in a variety of in vitro and ex vivo experimental systems. eEF2 phosphorylation is increased immediately after LTP-inducing tetanus in vivo (Belelovsky et al. 2005, 2007). It is important to note that eEF2 phosphorylation inhibits protein synthesis (Ryazanov and Davydova 1989); however, phosphorylated eEF2 promotes translation of specific mRNAs, e.g., dendritic up-regulation of Arc/Arg3.1 and CaMKIIα (Walden and Thach 1986; Scheetz et al. 2000; Park et al. 2008).

Increasing synaptic activity using bicuculline, a GABA_A_ receptor antagonist, results in a rapid NMDAR-dependent increase in p-eEF2 in cortical neurons (Lenz and Avruch 2005) and an increase after 24 h of stimulation in hippocampal neurons that is dependent upon mGluRs (Verpelli et al. 2010), suggesting that eEF2 is differentially regulated at the receptor level following rapid synaptic activity and in homeostatic plasticity. Furthermore, exposing cortical neurons to glutamate, NMDA, or AMPA, or hippocampal slices to NMDA results in a rapid increase in p-eEF2 (Marin et al. 1997; Belelovsky et al. 2005), and blocking NMDARs results in a decrease, in conjunction with rapid translation of *BDNF* and *Arc* (Autry et al. 2011), implying a role for phosphorylation of eEF2 in mediating glutamate receptor-mediated excitotoxicity (Hardingham and Bading 2010). In addition, blockade of NMDA receptor by the nonselective antagonist ketamine in the PFC has antidepressant effects and leads to increased synaptic protein synthesis via activation of the mTOR/S6K pathway and 4E-BP1, and prevents spine atrophy (Duman et al. 2012; Li et al. 2012). Furthermore, NMDA stimulation of hippocampal slices can also increase eIF4E phosphorylation in the CA1 region (Banko et al. 2004).

Stimulation of mGluRs alone also results in an increase in p-eEF2, and mGluR-stimulated LTD and Arc synthesis are deficient in eEF2K KO mice (Park et al. 2008). Interestingly, overexpression of eEF2K in the hippocampus results in deficits in L–LTP (Im et al. 2009). Thus, genetically manipulating eEF2K to increase or decrease its expression results in the disruption of hippocampal synaptic plasticity, suggesting that eEF2 is delicately poised to modulate synaptic strength.

Consistent with a complex role in modulating synaptic plasticity, eEF2 appears to be regulated in a compartmental-specific fashion in neurons. In an elegant series of experiments, Sutton et al. (2007) found that NMDAR-dependent miniature synaptic events in hippocampal neurons result in an increase in dendritic p-eEF2 that may act to restrain overall protein synthesis in this cellular compartment when neurons are in a resting state. In vivo, there are higher levels of p-eEF2 in synaptoneurosomal fractions than in total cell homogenate (Belelovsky et al. 2005, 2007). In dissociated hippocampal neurons, long-term treatment with bicuculline or tetrodotoxin has opposing effects on p-eEF2 in dendrites, which is important for dendritic BDNF protein synthesis but has no effect on p-eEF2 in the soma (Verpelli et al. 2010). Although it is not yet clear what the implications of compartment-specific modulation of eEF2 phosphorylation are for neuronal function, the fact that eEF2 can regulate both overall rates of protein synthesis and may regulate the synthesis of a particular subset of proteins (Park et al. 2008) suggests that the regulation of elongation has a multifaceted role in learning, memory, and synaptic plasticity.

## Translation regulation in the cortex: The taste case

One method for studying memory consolidation is a behavioral approach that exploits the robust novel taste learning in rodents. Three main paradigms are used in the context of taste learning: incidental taste learning (novel taste), conditioned taste aversion (CTA), and latent inhibition of CTA (LI-CTA), in all of which the molecular and neural substrates are well defined (Elkobi et al. 2008; Barki-Harrington et al. 2009; Gal-Ben-Ari and Rosenblum. 2011). CTA is a rapidly acquired task that involves the formation of an association between a novel taste (the conditioned stimulus; CS) and a malaise-induced state (the unconditioned stimulus; US) and is a protein synthesis-dependent task known to rely on the gustatory cortex and the amygdala (Rosenblum et al. 1993; Gal-Ben-Ari and Rosenblum 2011). In contrast, the latent inhibition paradigm consists of pre-exposure of the animal to a novel taste, followed by CTA, which by reducing taste neophobia allows addressing taste aversion per se. Recent studies using these behavior paradigms have demonstrated that the involvement of new protein synthesis in CTA is time limited (Merhav et al. 2006; Yefet et al. 2006; Merhav and Rosenblum 2008)

### mTOR and taste learning

The mTOR pathway plays an important role in various forms of synaptic plasticity, as has been demonstrated in several animal models (Casadio et al. 1999; Tang et al. 2002). Several correlative changes associated with novel taste or CTA paradigms involve proteins that are direct or indirect substrates of mTORC1 (Belelovsky et al. 2005). A recent study has shown that novel taste learning induces two temporal waves of mTOR activation in the gustatory cortex (GC) 15 and 180 min following taste learning (Belelovsky et al. 2009). These time-specific increases in mTOR phosphorylation coincide with similar time-specific increases in phosphorylation of S6K1, which constitutes one of the downstream targets of mTOR, and has been shown to be necessary for induction of protein-synthesis-dependent synaptic plasticity (Cammalleri et al. 2003).

Stereotaxic administration of rapamycin to the GC of naïve rats resulted in changes in phosphorylation (e.g., S6K1, eEF2) and protein levels (e.g., eEF1A) of mTORC1 targets, all peaking at 45 min following rapamycin administration. At the behavioral level, the stereotaxic administration of rapamycin to the GC impairs long-term taste memory, whether administered prior to the novel taste introduction, or afterward, prior to the second peak in mTORC1 activation as shown using the LI-CTA paradigm. This result demonstrates that the novel taste-learning-induced elevation in mTORC1 activation is necessary for the memory consolidation process. Biochemically, rapamycin administration resulted in reduced levels of PSD-95, a major postsynaptic scaffolding protein and known target of mTORC1 45 min following exposure to novel taste, in accordance with the time frame of rapamycin-induced changes of other mTOR targets examined (Belelovsky et al. 2009).

### eEF2 and taste learning

In addition to translation regulation at the initiation stage, control of elongation has also been found to play an important role in learning, memory, and synaptic plasticity, for which much evidence has come from taste learning and CTA studies. Following incidental taste learning, eEF2 phosphorylation increases within 20 min in the GC (Belelovsky et al. 2005; Gildish et al. 2012). In addition, transgenic mice that express a kinase-defective version of eEF2K and have significantly diminished levels of p-eEF2 are impaired in CTA learning, but not incidental taste learning, suggesting that the regulation of eEF2 phosphorylation is important specifically for associative taste memories (Gildish et al. 2012). The role of eEF2 phosphorylation in processing associative memory at the systems level was also examined using manganese-enhanced magnetic resonance imaging (MEMRI). eEF2K-deficient mice had a more diffuse accumulation of Mn^2+^ following CTA acquisition as compared with wild-type mice (Gildish et al. 2012), suggesting that the regulation of eEF2 participates in the regulation of brain activation patterns during associative memory formation.

In addition to taste learning, the regulation of eEF2 phosphorylation has also been examined following hippocampus-dependent contextual and hippocampus-independent cued fear conditioning (Phillips and LeDoux 1992; Logue et al. 1997). Interestingly, in contrast to CTA, fear conditioning results in a dramatic decrease in eEF2 phosphorylation within 0.5–2 h in both the hippocampus and amygdala of mice (Im et al. 2009). Furthermore, transgenic overexpression of eEF2K, which results in an increase in p-eEF2, impairs long-term contextual fear memory, but not cued fear memory. Thus, eEF2 is differentially regulated following various types of learning that involve distinct neural substrates, suggesting that the contribution of translation elongation regulation to learning and memory is quite complex.

## Translation regulation in the hippocampus

Research in the hippocampal formation has revealed a rich repertoire of protein-synthesis-dependent forms of synaptic plasticity. The major excitatory projections through the entorhinal–hippocampal circuits utilize glutamate as a neurotransmitter, yet the mechanisms of synaptic consolidation at each stage of the circuit may be uniquely tuned to mediate subregion-specific functions in learning and memory. The two regions of entorhinal–hippocampal circuit most studied in terms of translational control and synaptic plasticity are the CA1 region of the hippocampus proper and the dentate gyrus (DG). Below we summarize current knowledge of translational control of synaptic plasticity in the hippocampus, specifically contrasting and comparing findings from the CA1 and DG regions.

### mTORC1 signaling

The role of the mTORC1 pathway in translational control and long-term synaptic plasticity has been most extensively studied at Schaffer collateral-CA1 pyramidal cell synapses in acute hippocampal slices. The specific mTORC1 inhibitor rapamycin blocks development of late-phase CA1–LTP when the drug is applied during the period of high-frequency stimulation (Tang et al. 2002; Cammalleri et al. 2003). mTORC1-induced L–LTP has been recently demonstrated to be mediated by Wnt signaling and regulation of glycogen synthetase kinase-3, shown to function as an integrator of Akt and Wnt signals (Ma et al. 2011). mTORC1 signaling is also important in the establishment of protein-synthesis-dependent mGluR-dependent LTD in the CA1 region of hippocampal slices treated with the mGluR1/5 agonist 3,5-dihydroxyphenylglycine (DHPG) (Hou and Klann 2004). DHPG-induces phosphorylation of Akt (Ser473), mTOR (Ser2448), S6K (Thr389), ribosomal protein S6 (Ser 240/244), 4E-BP2 (Thr37/46), and synthesis of eEF1A in a rapamycin-sensitive manner (Hou and Klann, 2004, Banko et al. 2006, Antion et al. 2008a). Two downstream effector molecules of mTORC1, S6K and 4E-BP2, have also been implicated in synaptic plasticity (Hoeffer and Klann 2010). In hippocampal slices from 4E-BP2 knockout mice, ERK-dependent late LTP can be elicited by a single train of high-frequency stimulation (HFS), which in slices from wild-type mice generates only early LTP (Banko et al. 2005). 4E-BP2 knockout mice similarly exhibit enhanced protein-synthesis-dependent mGluR–LTD (Banko et al. 2006). However, the alterations in translation produced by knockout of 4E-BP2 are clearly disruptive for function, as Morris water maze and contextual cued fear conditioning is impaired, as is late LTP induced by standard stimulation protocols (Banko et al. 2005). In mice lacking either S6K1 or S6K2, normal protein-synthesis-dependent CA1–LTP was observed with repeated trains of HFS or θ burst stimulation. In addition, each of these strains demonstrated a different profile of alterations in behavior and synaptic plasticity (Antion et al. 2008b). LTP in the CA1 region of acute slices is associated with enhanced expression of dendritic eEF1A, which is encoded by mRNAs having a 5′ terminal oligopyrimidine (TOP) tract, in a rapamycin-sensitive manner (Tsokas et al. 2005), suggesting that the capacity of the translational machinery itself may be rate-limiting in synaptic plasticity. Moreover, mTOR signaling modulates translational capacity by driving ribosome production (rRNA synthesis), translation of mRNAs for ribosomal proteins, and translation of mRNAs for a number of translation factors (Iadevaia et al. 2008; Lempiainen and Shore 2009). The tuberous sclerosis complex (TSC) 1 and 2 gene products form a complex that inhibits the mTOR-activating brain-expressed ras homolog, Rheb. Disruption of TSC1 or TSC2 results in overexpression of mTOR, leading to abnormally rapid cell growth, hyperactivation of mRNA translation, and impaired synaptic plasticity. Clinically, this may be manifested by epilepsy, autism, intellectual disability, and self-injury (Gipson and Johnston 2012). In studies with TSC2 heterozygous knockout mice, facilitated late CA1–LTP was observed alongside deficient contextual LTM, which was rescued by systemic rapamycin injection (Ehninger et al. 2008). Hence, studies from genetically modified mice with mutant upstream negative regulators of mTORC1 or downstream targets of mTORC1 exhibit altered synaptic plasticity and memory. In a recent study, mTOR heterozygous knockout mice exhibited normal late CA1–LTP and LTM which was abolished by treatment with rapamycin (50 mg/kg) at a dose that is subthreshold for inhibition of LTP in wild-type controls (Stoica et al. 2011). This study indicates that submaximal levels of mTORC1 activation are sufficient for L–LTP generation.

Current understanding of translational control in DG synaptic plasticity stems mainly from studies of LTP of the medial perforant path input to granule cells of intact, anesthetized rats. As in the CA1, HFS-induced LTP in the DG is associated with increased Ser2448 phosphorylation of mTORC1 and downstream signaling to S6K and S6. Remarkably, while rapamycin blocks mTOR activation and signaling to S6, it has no effect on LTP maintenance during >4 h of recording. Moreover, eIF4F complex formation during LTP does not require release of 4E-BP2 from eIF4E, and rapamycin does not block initiation complex formation or the associated enhanced expression of Arc protein (Panja et al. 2009).

### ERK–MNK regulation in the hippocampus

ERK has emerged as a critical regulator of transcription and translation in protein-synthesis-dependent synaptic plasticity. ERK is critical for expression of both NMDA receptor-dependent (English and Sweatt 1997) and NMDA receptor-independent LTP in area CA1 (Coogan et al. 1999) and a variety of hippocampus-dependent memory formations. In hippocampal slices CA1–LTP is associated with ERK phosphorylation. Pharmacological inhibition of MEK, a kinase upstream of ERK, with PD098059 attenuates both early-LTP and late-LTP (English and Sweatt 1997). Hippocampus-related learning paradigms like contextual fear conditioning and Morris water-maze spatial learning are associated with phosphorylation of ERK, and inhibition of MEK with systemic administration of SL327 (Atkins et al. 1998) or local infusion of PD098059 in the hippocampal formation (Blum et al. 1999) inhibits memory formation.

A major substrate for ERK in translational control is MAP kinase-interacting kinase1 (MNK1) (Fukunaga and Hunter 1997). Activated MNK1 phosphorylates eIF4E on Ser^209^ (Shveygert et al. 2010) and enhanced eIF4E phosphorylation has been found in many studies of synaptic plasticity. For instance, incubation of mouse hippocampal slices with phorbol ester and forskolin resulted in ERK-dependent activation of MNK1 and increased eIF4E phosphorylation in the CA1 subregions (Banko et al. 2004). The biological functions of the MNK and the significance of MNK-mediated eIF4E phosphorylation have been controversial because MNK1/2 double-knockout (MNK–DKO) mice exhibit normal cell growth and development despite an absence of eIF4E phosphorylation (Ueda et al. 2004). However, synaptic plasticity and memory function have not been analyzed in these mice.

In the DG, BDNF–LTP, and HFS–LTP, both require ERK-dependent transcription and expression of the immediate early gene, Arc (Ying et al. 2002; Messaoudi et al. 2007). BDNF–LTP in the DG in vivo also resulted in rapid and transient eIF4E phosphorylation in DG lysates (Kanhema et al. 2006). In DG in vivo, HFS–LTP was inhibited by local PD098059 or U0126 infusion pre-HFS (Rosenblum et al. 2000; Panja et al. 2009). U0126 inhibited LTP-associated ERK phosphorylation, Arc synthesis and blocked translation initiation eIF4F complex formation (Panja et al. 2009). Infusion of U0126 at 10-min post-HFS effectively eliminated increased *Arc* mRNA and protein expression, suggesting Arc protein expression is intimately coupled to new mRNA production and indicating that ERK activation maintains *Arc* transcription for at least 10 min after LTP induction. The MNK1 inhibitor CGP57380 blocked HFS–LTP in the DG in vivo in parallel with a block of MNK–eIF4E signaling and Arc protein synthesis, while leaving *Arc* mRNA expression in the granule cells intact. Remarkably, MNK activation was required for loading of eIF4G onto eIF4E in eIF4F complex formation (Panja et al. 2009). Taken together, this suggests that MNK signaling, not mTORC1 signaling, underlies initiation complex formation, Arc synthesis, and LTP consolidation in the DG. Although the impact of eIF4E phosphorylation on translation efficiency is debated (Sossin and Lacaille 2010), its phosphorylation via MNK may result in increased translation of proteins crucial for LTP maintenance in the DG.

### eEF2 regulation in the hippocampus

The eEF2 phosphorylation state is modulated during synaptic plasticity in the CA1 region and DG. In the CA1 region of acute hippocampal slices, chem-LTP is associated with increased phosphorylation of eEF2 at 1-h post treatment, coincident with inhibition of de novo total protein synthesis and enhanced expression of Arc (Chotiner et al. 2003). mGluR-induced LTD in the CA1 region of hippocampal slices requires local synthesis of Arc from preexisting mRNA (Waung et al. 2008), and this synthesis is mechanistically linked to eEF2 phosphorylation (Park et al. 2008). Upon mGluR activation, the receptor-associated eEF2K dissociates from mGluRs and phosphorylates eEF2. It is speculated that p-eEF2 inhibits FMRP, which binds to *Arc* mRNA at the synapse (Zalfa et al. 2003) and inhibits its translation (Bear et al. 2004).

BDNF–LTP in the DG in vivo is associated with ERK-dependent phosphorylation of eEF2 in DG lysates (Kanhema et al. 2006). However, BDNF treatment of synaptoneurosomes did not increase eEF2 phosphorylation, though eIF4E phosphorylation and rapid synthesis of Arc and CaMKIIα proteins were still observed (Yin et al. 2002; Schratt et al. 2004; Kanhema et al. 2006). This suggests a compartmental-specific differential regulation of eEF2 at the synapses in response to BDNF. During HFS–LTP in the DG in vivo*,* eEF2 is rapidly elevated and remains elevated for at least 4 h (Panja et al. 2009). NMDAR blocked by AP5 inhibited LTP induction and Arc expression without affecting eEF2 phosphorylation at 15 min and 2 h post-HFS, though phosphorylation of eEF2 at 4 h was blocked, indicating delayed NMDAR-dependent phosphorylation of eEF2. However, rapamycin blocked eEF2 phosphorylation without affecting LTP or Arc expression (Panja et al. 2009). It is therefore unclear at present what function eEF2 phosphorylation may play in LTP in the DG. The sustained nature of eEF2 phosphorylation and its delayed regulation by NMDAR points to involvement in late LTP. Alternatively, eEF2 phosphorylation may be linked to other forms of plasticity such as late LTD (Park et al. 2008).

## Differences between CA1 and DG

These findings indicate fundamental differences in translational control of synaptic plasticity between CA1 and DG. mTORC1 signaling is required for CA1–LTP, mGluR–LTD, and hippocampal-dependent learning (Hoeffer and Klann 2010). In contrast, induction and maintenance of DG–LTP is rapamycin-insensitive (Panja et al. 2009), despite the fact that mTOR signaling to S6 is activated and effectively blocked by rapamycin. In the CA1 region, mTOR phosphorylates 4E-BP2 and regulates eIF4F formation (Banko et al. 2005), while in the DG, ERK signaling to MNK regulates eIF4F, Arc synthesis, and LTP consolidation (Panja et al. 2009). To date, the mouse mutants that have been used to dissect translational control of synaptic plasticity in the CA1 region have not been applied to the DG, and the potential role of ERK–MNK in eIF4F formation in CA1 has not been explored in detail. The regional differences in translational regulation could to some extent reflect the use of the hippocampal slices for studies of CA1-LTP and anesthetized rats for studies on the DG. However, this seems unlikely given the convergence of pharmacological and genetic approaches applied to the analysis of mTORC1 function in CA1, amygdala, and neocortex. It is therefore intriguing to consider that different styles of translation in the CA1 and DG may generate distinct patterns of protein expression that contribute to subregion-specific functions of the DG (pattern separation) and CA1 region (temporal pattern associations) in memory processes (Tsien et al. 1996; Rolls and Kesner 2006; McHugh et al. 2007; Niewoehner et al. 2007).

## Regulation of miRNA activity in neuronal plasticity and memory storage

The diversity, target specificity, and reversible regulation of microRNAs in response to environmental cues make them ideal modulators of local protein synthesis in diverse cell types and biological contexts. Many new brain-specific microRNAs have appeared with vertebrate and primate evolution and roles for specific microRNAs in neurogenesis, dendritic spine morphogenesis, synaptic regulation, plasticity, and memory storage have been demonstrated (Vo et al. 2005; Krichevsky et al. 2006; Schratt et al. 2006; Fiore et al. 2008; Rajasethupathy et al. 2009; Gao et al. 2010; Mellios et al. 2011; Siegel et al. 2011; Tognini et al. 2011). Recent work has revealed the evolutionary divergence in neuronal miRNA expression that may account for brain-region-specific differences in gene expression between humans, chimpanzees, and macaques (Hu et al. 2011).

Understanding how microRNA function is regulated by neural activity is a major goal of current research. MicroRNA activity can be modulated by altering the abundance of mature miRNA relative to target, for instance, through activity-dependent transcription, processing, and metabolism of miRNAs (Vo et al. 2005; Kosik 2006; Impey et al. 2010; Krol et al. 2010; Wibrand et al. 2010; Huang et al. 2012; Saba et al. 2012). This type of mechanism is well established in microRNA-mediated effects on neuronal differentiation or development. However, consolidation of LTP and LTD starts rapidly and involves regulation of the local protein synthesis in dendrites. miRNA mechanisms should be correspondingly rapid, spatially restricted, and perhaps reversible. Current evidence suggests that synaptic activity may regulate miRNA activity through changes in local miRNA turnover and through modulation of miRISC proteins (e.g., Huang et al. 2012).

MicroRNA-124 is one of the most studied brain-specific miRNA and when overexpressed in cervical carcinoma cells (HeLa), miR-124 imparts a neuronal phenotype. miR-124 down-regulates several antineuronal genes, including *REST*, a transcriptional repressor of neuronal genes including miR-124 itself (Conaco et al. 2006). It also targets *Sox9*, among others, suppressing a glial phenotype, and promoting neuronal phenotype in subventricular zone stem cells (Cheng et al. 2009). miR-124 is highly conserved across species and is expressed in *Aplysia* where it controls serotonin-induced synaptic facilitation (LTF) through repression of CREB. With several putative CREB-binding sites in its promoter, miR-124 might be under control of a feedback mechanism (Rajasethupathy et al. 2009).

MicroRNAs are implicated in dendritic arborization and spine morphogensis during activity-dependent neuronal development. Activity-dependent transcription of CREB-dependent miR-132 is both necessary and sufficient for neurite outgrowth (Vo et al. 2005). Like many neuronal CREB targets, miR-132 is highly induced by BDNF. By inhibiting expression of the Rho GTPase-activating protein p250GAP, miR-132 stimulates actin remodeling and dendritic morphogenesis of cortical and hippocampal neurons (Wayman et al. 2008; Impey et al. 2010).

The involvement of microRNAs in shaping the dendritic spine was first described for miR-134. Overexpression of miR-134 in hippocampal neurons reduces dendritic spine size at least in part by repression of Lim-domain-containing protein kinase 1 (LIMK1), a major regulator of actin cytoskeletal dynamics. Application of BDNF relieved LIMK1 from miR-134-mediated repression, suggesting neuronal activity-induced reversal of repression (Schratt et al. 2006). A similar function has later been described for miR-138 through its regulation of acyl protein thioesterase (APT1) mRNA.

A bidirectional response on microRNA expression has been shown with both LTP and LTD induction. Park and colleagues used chemical LTP and metabotropic glutamate receptor-dependent LTD (CA1) in hippocampal slices to study plasticity-induced changes in microRNA expression (Park and Tang 2009). They observed rapid (15 min) and dynamic changes in expression profiles. On the other hand, high-frequency stimulation of the medial perforant pathways in the dentate gyrus (DG) in vivo resulted in bidirectional microRNA changes at a later time point (2 h) (Wibrand et al. 2010). In the adult DG, mature miR-212 and miR-132 were up-regulated and miR-219 down-regulated. Surprisingly, block of NMDA receptor-dependent LTP led to enhanced expression of these mature miRNAs. Primary and precursor transcripts for miR-212 and miR-132 were very strongly induced by HFS, but this up-regulation was not modulated by NMDA receptor block, though it was completely inhibited by mGluR block. Precursor levels for miR-219 were unchanged during LTP, yet mature miR-219 exhibited an NMDA receptor-dependent down-regulation. These results suggest that (1) mGluR and NMDARs interact in the regulation of the miRNA transcription and biogenesis and (2) that some mature miRNAs are subject to NMDA receptor-dependent destabilization and decay. Recent studies have also demonstrated regulation of AMPA receptors by miR181a and mir181b (Saba et al. 2012). The control of miRNA-124 function by neurotransmitter serotonin in *Aplysia* and relief of miR-134-mediated repression of LimK1 by BDNF are classic examples of regulation of miRNA function upon neuronal activity (Schratt et al. 2006; Rajasethupathy et al. 2009). These also indicate the control of miRNA activity via classical signaling pathways. Other possible mechanisms of miRNAs include degradation/decay and RNA editing. Though very little is known regarding this, evidence indicates that neuronal activity is often accompanied by high miRNA turnover (Krol et al. 2010; Kye et al. 2011). Understanding mechanisms of miRNA turnover in neurons could provide insight into the regulation of miRNA function.

Lugli et al. (2005) demonstrated a post transcriptional mechanism of regulating miRNA activity. In synaptosomes, activity-dependent cleavage of calpain-induced Dicer activity resulted in enhancement of miRNA production. Neuronal activity could also modulate miRNA function through the miRISC components. Neuronal stimulation induced inactivation and complete removal of miRISC component Armitage, as observed in *Drosophila*, and ubiquitin-mediated degradation of MOV10 in depolarized neurons in culture, open the possibility of regulation of miRNA function via miRNA-associated protein factors (Ashraf et al. 2006; Banerjee et al. 2009). In flies, degradation of the RISC-associated RNA helicase, Armitage, promotes synaptic protein synthesis during long-term memory (Ashraf et al. 2006; Kosik 2006; Winter et al. 2009). Similarly, proteasomal degradation of an orthologous RNA helicase, MOV10 protein, mediates derepression of miR-26 in dendrites of cultured hippocampal neurons (Banerjee et al. 2009).

MicroRNAs often regulate and are regulated via RNA-binding proteins. MicroRNA-134 regulates activity-dependent dendrite remodeling through Pumilio2, a RNA-binding protein that regulates neuronal mRNA translation (Fiore et al. 2009). In another study, virus-mediated overexpression of miR-134 in the CA1 region of hippocampus in mice abrogated LTP and impaired long-term memory formation during contextual fear conditioning (Gao et al. 2010). These studies along with those done in *Aplysia* long-term facilitation indicate a role of miRNA in synapse function. A recent finding indicated that overexpression of miR-132 decreased spine density, but increased average spine width. In the same study, miR-125b overexpression led to longer and thinner spines. Both of the miRNAs were shown to interact with the RNA-binding protein FMRP, and in the absence of FMRP, the effect of the miRNA was abolished (Edbauer et al. 2010). Recently, interaction of miR-125a with FMRP has been shown to regulate reversible translation of PSD-95 mRNA in dendrites (Muddashetty et al. 2011). Activity-dependent phosphorylation of FMRP was shown to modulate this interaction and hence affect the miRNA function.

Although the involvement of microRNAs in neuronal differentiation, synapse morphology, and plasticity is well established in vitro*,* recently it has been shown that microRNA function could be directly linked to learning and memory in vivo. The first study addressing this question in vivo has recently been published (Konopka et al. 2010). *Dicer 1*, a key gene for the biogenesis of mature microRNAs was conditionally deleted in the forebrain neurons of adult mice. The lack of mature microRNAs in the targeted cells resulted in a significant improvement in different learning tasks such as spatial memory in the Morris water maze and contextual fear conditioning. Neurons exhibited long filipodia-like spines and enhanced post-tetanic potentiation (though LTP was not affected). It was proposed that removal of mature microRNAs from the forebrain neurons might facilitate translation of synaptic mRNAs. Indeed, the investigators were able to show increased levels or activity of several proteins with the ability to affect plasticity, known to be translated in dendrites (BDNF, PSD-95, Glur1/GluR2, and MMP-9).

miR-132 has, as previously mentioned, well-characterized effects on dendritic spines in vitro. Transgenic animals overexpressing miR-132 in their forebrain neurons show increased density of dendritic spines, decreased levels of MeCP2, and deficits in novel object recognition (Hansen et al. 2010). Another microRNA that affects the morphology of dendritic spines both in vitro and in vivo is miR-134 (Schratt et al. 2006). Christiansen and colleagues used recombinant adeno-associated virus (rAAV) to overexpress miR-134 in the adult mouse brain and reported a negative role of miR-134 in dendritic arborization of cortical layer V pyramidal neurons (Christensen et al. 2009).

Recently, a new molecular pathway describing the regulation of neuronal plasticity and memory by miR-134 has been described (Gao et al. 2010). The investigators showed that the cAMP-response binding protein (CREB) was post-transcriptionally regulated by miR-134 and that the expression of miR-134 is negatively regulated by SIRT1 through a repressor complex. In SIRT1 knockout mice, up-regulation of miR-134 reduced the amount of CREB protein and, consequently, also of its target gene, BDNF. As a result, impaired synaptic plasticity and defects in learning and memory were observed. That was the first time the role of SIRT1 in higher brain function was shown. Interestingly, the translation of SIRT1 mRNA is regulated by two members of the miR-34 family, miR-34a and miR-34c (Aranha et al. 2011; Zovoilis et al. 2011). Recent work elegantly showed a role for miR-34c in contextual fear conditioning, and identified SIRT1 as a decisive target for this regulation (Christensen et al. 2009). miR-34a regulates, on the other hand, SIRT1 in the context of neuronal stem-cell differentiation (Aranha et al. 2011). A recent study has identified miR-34a as one of three miRNAs that cooperates in regulation of the Arc 3′ UTR and which inhibits endogenous Arc protein expression in neurons (Wibrand et al. 2012).

## Computational approach for modeling molecular mechanisms underlying learning and memory

As described in the previous sections, many different biological processes make important contributions to learning and memory regulation at the molecular level. In order to fully understand these processes, assimilation of the many different types of experimental data that describe the phenomenon is required (Ghosh et al. 2011; Tenazinha and Vinga 2011). Crucially, this process necessitates experimental paradigms that are well understood, data rich, and span the multiple levels of genetic control described. The technologies that allow for the routine assay of multiple molecular types and their biochemical modifications on a genomic scale (e.g., RNA-seq, ChIP-seq, miRNA and mRNA arrays, high resolution proteomics, protein phosphorylation, and protein–protein interaction data) now exist, but methods for their effective modeling both temporally and by cellular compartment are not yet fully developed. Therefore, efforts are being made to create principled and biologically meaningful representations of these large-scale data in models that are flexible enough to accommodate both the raw data itself and preexisting biology/neuroscience established knowledge.

Systems Biology modeling has been widely used in biology for many years; it frequently comprises just a single data type (for example, mRNA level or protein concentration) or uses small numbers of molecules or canonical pathways and rarely takes spatial constrains into consideration. More recently, integrative methods have begun to overlay multiple data sources onto these models, for example, visualizing mRNA expression data in the context of protein-interaction networks (Alcaraz et al. 2012; Li et al. 2012) or proteomic data (Hallock and Thomas 2012), but these methods of data integration do not implicitly model the relationships between the different data types, and the functional insight obtained is limited.

### Toward dynamic integrative models of synaptic plasticity regulation

Synaptic plasticity is considered the molecular and cellular correlate of learning and memory (Lynch 2004) and is a biological process for which there is a wealth of molecular and physiological data, some of which is summarized above. How do we merge this understanding with current systems biology models of molecular cognition? A number of recent studies have modeled the synaptic proteome focusing on its composition and function, typically representing the relationships between members in protein–protein interaction networks (PINs) where network membership is determined by proteomic profile and connectivity by the propensity for constituent proteins to physically interact in vitro (Collins et al. 2006; Pocklington et al. 2006; Fernandez et al. 2009). These networks have been used to dissect out functional modules of the postsynaptic density, map disease associations, and assess the evolutionary conservation of function.

To take the computational approach to a higher level, modeling approaches have moved beyond static representations of synaptic plasticity by both changing the way in which the process is modeled and by acquiring the data necessary to do so (Kotaleski and Blackwell 2010). Across the processes of memory acquisition, consolidation, and retrieval regulation can be found at the transcriptional (Alberini 2009), post-transcriptional (Bredy et al. 2011), translational (Costa-Mattioli and Sonenberg 2008), and post-translational (Routtenberg and Rekart 2005) levels in addition to temporal and compartment-specific components. Recently, several rule-based methods have been developed that allow the description of complex molecular systems with essentially no limit to the type or nature of the molecular entities or relationships defined (Bachman and Sorger 2011). The most widely used examples of these are “Kappa Language” (Feret et al. 2009) and the “Network-Free Stochastic Simulator” (NFSim) (Sneddon et al. 2011), which are capable of integrating static, stochastic, and kinetic representations within the same model. Our understanding of the molecular mechanisms controlling memory consolidation as described in the preceding sections is dominated by two key types of biochemical reaction; binding (protein–protein, miRNA–RNA, and protein–DNA) and site-specific protein phosphorylation. Using rule-based languages, these reactions can be encoded in complexes and pathways that more realistically reflect the underlying biology. Further, the qualitative and quantitative measurements derived from transcriptomic, proteomic, phospho-proteomic, and sequence analyses can be used to parameterize models and allow the simulation and perturbation experiments necessary to both understand consolidation at a systems level and seed model refinement by comparing real and simulated data.

The first Kappa language model of the core postsynaptic density complex using domain interaction and protein availability data has recently been reported (Sorokina et al. 2011) demonstrating the feasibility and utility of rule-based models in this domain. Models such as this can be extended to describe the mechanisms of memory consolidation including translation, miRNA, and phosphorylation-mediated regulation. By modeling at different time points and in different parts of the brain (for example, DG and CA1) we can learn more about the regulatory specializations that define the different phases of learning and memory.

Concluding remarks: As described above, in recent years we have gained a lot of new information about the details of memory and synaptic plasticity consolidation at the level of translation regulation. The clear differences described above, between different brain structures such as cortex and hippocampus, as well as the differences between different subfields of the hippocampus itself, shed new light on the fine details of consolidation and possibly the function of these brain areas. Clearly, new biological methods are needed to better reveal the process of consolidation at the single neuron or subneuron level. These new methods together with new computational models for neurobiology, promise to allow, for the first time, the simulation of effects that span multiple levels of biology from the molecular to the physiological. These new systems biology and molecular approaches may form the first effective bridge between molecular systems biology and the neuroscience of learning and memory.
